# A hydroponic system for facilitating observation of plant virus infection in roots

**DOI:** 10.1080/15592324.2026.2644050

**Published:** 2026-03-16

**Authors:** Guoliang Chen, Yuchen Lei, Haijuan Wang, Yan Lu, Hongyou Zhou, Mingmin Zhao

**Affiliations:** a College of Horticulture and Plant Protection, Inner Mongolia Agricultural University, Hohhot, People's Republic of China; b Key Laboratory of the Development and Resource Utilization of Biological Pesticide in Inner Mongolia, Hohhot, People's Republic of China

**Keywords:** Hydroponics system, tobacco mosaic virus (TMV), root development, viral infection

## Abstract

Plant viruses usually spread from the infection site to upper young leaves through plant phloem along with the plant nutrient solution flow. To track the infection status of plant viruses in root systems, we established an independent and controllable hydroponic system for growing single **N*. benthamiana* plants. The infection of tobacco mosaic virus (TMV-GFP) in roots of *N. benthamiana* grown in hydroponic system was evaluated. The results showed that the seed germination of *N. benthamiana* in hydroponic system on day 3 after sowing. By day 5, the hypocotyls had elongated and the cotyledons had expanded. By day 7, all seeds were germinated. Hydroponic seedlings showed cotyledon expansion at 3 d but slow leaf development, whereas soil-grown seedlings exhibited cotyledon expansion at 6 d with rapid leaf growth. Hydroponic plants were significantly taller than soil-grown plants at the same growth stage. After TMV-GFP infection, root growth of *N. benthamiana* was significantly inhibited, characterized by slowed lateral root development, thinner roots, and reduced root numbers. Both fluorescence intensity and viral accumulation of TMV-GFP in the roots of hydroponic plants increased significantly than those of plants in soil. In summary, this hydroponic system displayed several advantages for viral infection study in roots, such as non-destructive, real-time observation of roots and allows for single-factor control, effectively avoiding interference from soil cultivation. It might be a convenient technique for studying plant root–virus interactions.

## Introduction

Roots, as vital organs for water and nutrient uptake in plants, are closely interconnected with the soil environment. Their physiological functions and structural characteristics directly determine plant growth status and stress resistance.^1-3^ Various plants have also evolved specialized root adaptations to thrive in diverse environments. For instance, leguminous plants form root nodules to enable biological nitrogen fixation.^4^ Desert plants frequently develop root cuticles to minimize water loss and enhance drought tolerance,^5^ while crops such as radishes, carrots, and beets accumulate nutrients in their roots, forming enlarged storage organs.^6^^,^^7^

Hydroponic technology enables plants to be free from the dependence on soil and provides an artificially controllable liquid environment for root growth. By immersing roots in a transparent liquid medium, it becomes possible to observe the dynamic growth process of plant roots and to measure morphological parameters accurately. This could thereby avoid the damage and observational biases associated with root excavation in soil-based cultivation.^8^^,^^9^ The hydroponic environment allows precise control of factors such as nutrient supply, dissolved oxygen, pH, and temperature, facilitating the study of how individual factors affect root physiological metabolism.^10-12^ In addition, this approach also provides controllable conditions for investigating root metabolic mechanisms and stress physiology, avoids the interference from multiple interacting factors typical in soil cultivation, and improves the scientific validity and reliability of research outcomes.

Plant viruses cause a serious threat to crop production in the whole world. In general, most plant viruses can infect a wide range of crops, leading to reduced yields and diminished quality, thereby endangering food security and sustainable agricultural development.^4^^,^^13-15^ For instance, Tobacco mosaic virus (TMV-GFP) can cause more than 80% yield loss in tomatoes, severely impacting agricultural ecosystems and supply chains in major production areas.^16^ The plant roots are important for systemic infection of plant viruses. Viral infection disrupts the physiological and metabolic balance of the root system, inhibiting its growth and function. After entering root cells, the virus interferes with nucleic acid and protein synthesis, resulting in stunted root growth, reduced morphological parameters, damage to the epidermal barrier and vascular tissues, decreased root activity and nutrient uptake efficiency, and ultimately causing abnormalities in the above-ground parts of the plant.^17^

In this study, a hydroponic system was constructed, in which the individual root systems of *N. benthamiana* plants were grown in relatively independent and controllable environments through the interlocking design of the bases. Using TMV labeled with green fluorescent protein (GFP) as the inoculum, we inoculated plants (with 4–5 leaves) growing in hydroponic system to observe the viral infection status in the roots. The impact of viral infection on the root systems of individual plants and the accumulation of the virus were analyzed. Compared with traditional hydroponics, hydroponic system could track the dynamic infection processes of TMV in intact roots. In addition, this technique has distinct advantages, such as non-destructive observation of viral infection in roots; maintain the internal physiological and biochemical processes of the root unaffected; avoiding soil contamination; improving research accuracy and repeatability.

## Materials and methods

### Plant materials

*N. benthamiana* seeds were preserved in our laboratory.

### Soil parameters

The soil substrate was purchased from Mengfei Biotechnology Co., Ltd. The physicochemical properties were as follows: pH 6.51, EC 1.855 mS/cm, TDS 1187 ppm.

### Sterilization of *N. benthamiana* seeds

Seeds were placed in a 2 mL centrifuge tube in a laminar flow cabinet. 1 mL of sterile water was added, and the tube was vortexed for 30 s and centrifuged at 4000 r/min for 1 min. The supernatant was removed using filter paper. Then, 1 mL of 75% ethanol was added; the tube was vortexed for 30 s and centrifuged at 4000 r/min for 1 min again before the waste liquid was discarded. Subsequently, 1 mL of sterile water was added, and the tube was vortexed for 1 min, centrifuged at 4000 r/min for 2 min, and the supernatant was removed. Next, 1 mL of a 10% NaClO solution (10 g NaClO a 100 mL water) was added. After overtaxing for 1 min, the mixture was standing for 5 min, and then the waste liquid was discarded. Finally, 1 mL of sterile water was added, and the seeds were vortexed for 30 s, and centrifuged at 4000 r/min for 1 min, and the supernatant was discarded. This washing step was repeated three times.

### Preparation of culture media

Preparation of 1/2 MS solid medium: Add 1.1 g of MS powder (purchased from Beijing Coolaber Co., Ltd.), 0.55 g of agar, and 0.6 g of sucrose into a 1 L conical flask containing 500 ml of distilled water, followed by autoclaving at 121 °C for 15 min.

Preparation of 1/4 MS liquid medium: Add 0.55 g of MS powder (purchased from Beijing Coolaber Co., Ltd.) into a 1 L conical flask containing 500 ml of distilled water, followed by autoclaving at 121 °C for 15 min. The physicochemical properties of the prepared nutrient solution were as follows: pH 5.63, EC 2.51 mS/cm, TDS 1606 ppm.

### Hydroponic device design

The hydroponic device for seed germination is designed with a hydroponic box and a hydroponic cap (with 1 holes with 4 mm in diameter) covered at the top of the hydroponic box. 1/2 MS culture medium was added in the hydroponic cap.

The seedlings with two leaves together with the hydroponic cap were transferred into a bottomless PP tube with a corresponding cap. The PP tube is 9 cm in length, and its cap is drilled with a 10 mm hole, forming an interlocking structure with the hydroponic cover.

When the plants grow to the 4–5 leaves, the seedlings, hydroponic cap, and PP tube cap are transferred into a flat-bottomed test tube. The opening of the flat-bottomed test tube has the same diameter (30 mm) as the PP tube cap, forming an interlocking structure.

### Hydroponic cultivation of *N. benthamiana* plants

Sterilized seeds were sown into the center of a base containing 1/2 MS solid medium. The plants were then transferred to a greenhouse and grown until the fourth young leaf had fully expanded. At this stage, the plants, together with the base, were moved into a hydroponic box with pre-drilled holes. Once the plants reached the 4–5 leaf stage, they were transferred to flat-bottomed test tubes for individual cultivation. Three biological replicates were used for each treatment.

### GFP fluorescence imaging of viral symptoms

Viral symptoms were observed and recorded at 3 d post-inoculation (dpi). GFP fluorescence imaging was performed using a GFP imager (a LUYOR-3104 handheld nondestructive UV lamp) with a wavelength of 360–370 nm. The root systems of the plants were collected and stored at −80 °C.

### Inoculation of TMV-GFP

A single colony of Agrobacterium tumefaciens strain C58C1 harboring the TMV-GFP infection clone was inoculated into liquid LB medium containing the corresponding antibiotic and incubated overnight at 28 °C with shaking at 180 r/min. Successful transformation was confirmed by PCR amplification using the bacterial culture. The bacterial suspension was then transferred to a 5 mL centrifuge tube and centrifuged at 4000 r/min for 15 min. After discarding the supernatant, the collected bacterial pellet was resuspended in an induction buffer (0.5 mmol/L MES, 1 mmol/L MgCl_2_, 0.1 mmol/L acetosyringone). The suspension was left at room temperature for 5 h and then diluted to an OD_600_ of 1.0 for inoculation. The fourth or fifth leaves of *N. benthamiana* plants were selected and used for viral inoculation with a 1 mL sterile needleless syringe.

### Detection of viral accumulation by western blot

The samples were ground into a powder in liquid nitrogen. After weighing, a 2× volume of protein lysis buffer was added, and the mixture was vortexed thoroughly. This was followed by incubation at 95 °C for 10 min and centrifugation at 12,000 r/min for 10 min at 4 °C. Three replicates were used for each samples.

The prepared protein samples were used for western blot analysis to detect viral accumulation Gels were prepared using a 12% stain-free gel kit (BIO-RAD). HRP-conjugated GFP recombinant rabbit monoclonal antibody (Huaan Biotech, Cat. No.: ET1702-69, clone JF51-05, RRID: AB_3070329) was used at a dilution of 1:2500 for Western blot detection of GFP-fused proteins. The antibody was directly conjugated with HRP, and ECL substrate was used for signal development. Bands of rubisco were used as the loading control. The prepared gels were placed into the electrophoresis apparatus, and electrophoresis was performed until the bromophenol blue tracking dye approached the bottom of the gel. The proteins were then transferred onto a nitrocellulose (NC) membrane. After transfer, the NC membrane was immersed in a blocking solution (1.5 g skimmed milk powder in 30 mL TBST) and blocked at room temperature for 2 h. The blocking solution was rinsed off the membrane with 1x TBST buffer. The membrane was then incubated with the primary antibody at 4 °C overnight. The following day, the membrane was removed and washed twice with 1× TBST buffer for 10 min each, followed by one wash with 1× TBS buffer for 10 min. The washed membrane was then incubated with the secondary antibody for 1 h. After incubation, the membrane was washed twice with 1× TBST buffer for 10 min each and once with 1× TBS buffer for 10 min. Finally, the membrane was incubated in an ECL developing solution, and the protein bands were visualized. using the Odyssey® Fc Dual-Mode Imaging System, which utilizes near-infrared dual-color laser and chemiluminescence detection.

### Statistical analysis and data quantification

The band density of Western blot was quantified using Image J software (National Institutes of Health, USA). All experimental data were plotted and statistically analyzed using GraphPad Prism 10.1.2 (GraphPad Software, San Diego, CA, USA). Data were presented as the mean ± standard deviation (SD) of three independent biological replicates, and students *t*-test was used to determine significant differences between groups, with *P *< 0.01 considered statistically significant.

## Results

### The design of hydroponic system for *N. benthamiana* plants

To facilitate the observation of viral infection in plant roots, we designed a hydroponic system for plant growth, particularly for *N. benthamiana.* Basically, this method contains following steps: Step 1, disinfection of *N. benthamiana* seeds; Step 2, preparation of the culture medium; Step 3, germination of tobacco seeds; Step 4, hydroponic cultivation of tobacco seedlings; Step 5, replacement of the culture medium; Step 6, inoculation of tobacco mosaic virus. When the tobacco plants reach the four-leaf stage, the plants along with the culture medium is transferred to a 50 ml centrifuge tube without a bottom to continue hydroponic cultivation. During the cultivation period, avoid light to prevent algae growth and replace the 1/4 MS liquid culture medium every 5 d.

**Figure 1.**
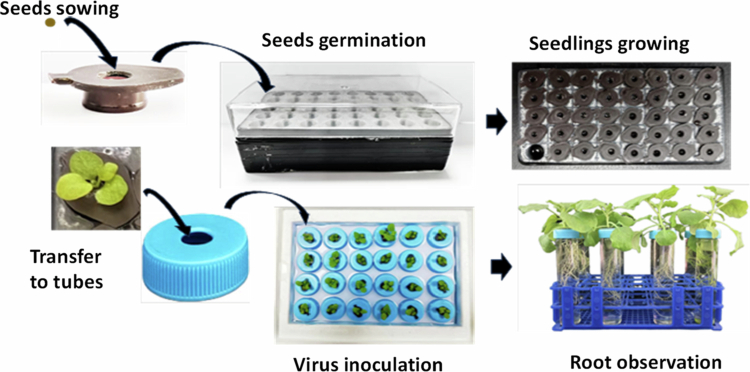
Schematic of the hydroponic system for *N. benthamiana* plants.

This hydroponic system utilizes the cooperation between different-sized substrates and hydroponic boxes to significantly improve the need for root integrity and reduce the damage to the lateral roots of *N. benthamiana*. The structural design of the cultivation device facilitates the complete extraction of the root system of plant, which effectively ensures the smooth progress of the research on root-related experiments (Figure 1).

### Seeds germination of *N. benthamiana* in hydroponic system

**Figure 2.**
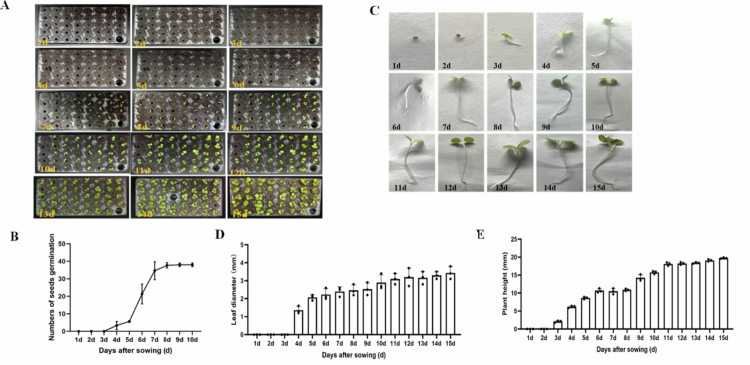
Germination dynamics of *N. benthamiana* seeds under hydroponic conditions. (A) Global status of seed germination; (B) Number of germinated seeds; (C) phenotype of single plants; (D) Leaf diameter; (E) plant height.

A total of 40 holes in hydroponic system were prepared, with one hole used for adding nutrient solution. Thus, a total of 39 seeds were then placed individually in the center of the holes on the culture medium base. The germination boxes were then transferred to a greenhouse for cultivation. Seed germination and seedling growth were recorded every day (Figure 2A).

The results showed that radicles emerged from the seed coats on day 3 after sowing. By day 5, the hypocotyls had elongated and the cotyledons had expanded. By day 7, all seeds were germinated (Figure 2B). We also observed seed development continuously for 15 d (Figure 2C). Seedlings were sampled daily (three plants per time point) to measure leaf diameter and plant height. The results showed hydroponically-grown seeds displayed rapid germination and growth (Figure 2D,E).

### Seeds germination of *N. benthamiana* in soil

**Figure 3.**
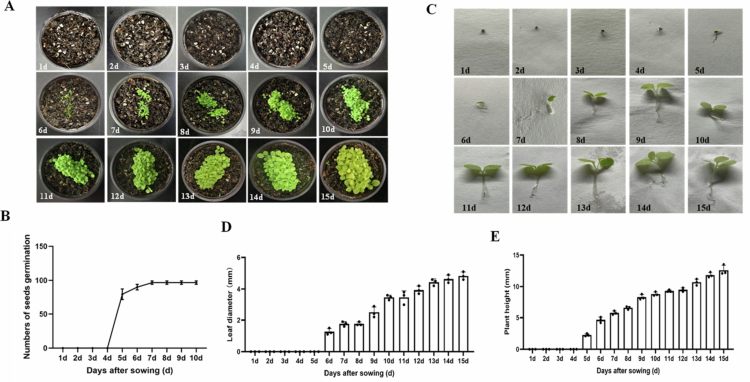
Seeds germination trend of *N. benthamiana* in soil. (A) Germination status of seeds; (B) Number of germination seeds; (C) phenotype of single plants; (D) Leaf diameter; (E) plant height.

Soil substrate seedling culture was free of spatial constraints, and 100 sterile seeds were selected for sowing to facilitate statistical analysis of germination data. The seeds were placed in a 5 mL centrifuge tube with 2  mL of distilled water, then evenly sown into the soil substrate using a dropper. The seedling pots were transferred to a greenhouse for cultivation. Seeds germination as well as seedling growth was monitored continuously. The results showed that approximately 82 seeds had radicles protruding from the seed coat by day 5 after sowing (Figure 3A,B). By day 6, around 90 seeds exhibited elongated hypocotyls and expanded cotyledons. By day 7, about 99 seeds had completed germination. The development of young leaves was also recorded. Cotyledons were fully expanded by day 10, the first true leaf was fully expanded by day 12, and the second true leaf was fully expanded by day 14 (Figure 3C). Statistical analysis of leaf diameter revealed soil-grown seedlings exhibited cotyledon expansion at 6 d with rapid leaf growth (Figure 3D). The plant height was gradually increasing and reach to highest at 15 d (Figure 3E).

To compare the germination of *N. benthamiana* seeds under soil and hydroponic conditions (Figures 2 and 3), hydroponically grown seeds displayed rapid germination and fast growth, while soil-grown plants showed slow germination at the early stage followed by rapid growth at later stages. In addition, seedling sampling under hydroponic conditions better prevented root damage compared with soil culture. Statistical analysis of leaf diameter revealed a gradual increase over time under both conditions. Particularly, hydroponic seedlings showed cotyledon expansion at 3 d but slow leaf development, whereas soil-grown seedlings exhibited cotyledon expansion at 6 d with rapid leaf growth. Statistical analysis of plant height showed similar growth trends between the two groups, and hydroponic plants were significantly taller than soil-grown plants at the same growth stage.

### Effects of TMV-GFP infection on *N. benthamiana* plants grown in hydroponic system

**Figure 4.**
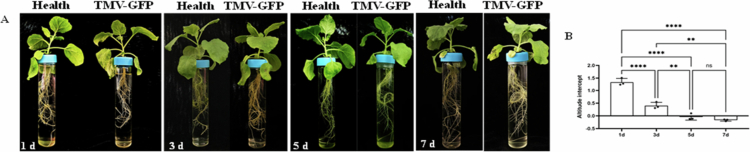
Effects of TMV-GFP infection on the root system. (A) Symptoms of TMV-GFP infection; (B) Changes in transpiration rate; ** *P *< 0.01, **** *P *< 0.0001, ns indicates no significant difference.

Seedlings of *N. benthamiana* at the 4–5 leaf stage were individually transplanted into flat-bottomed tubes and inoculated with TMV-GFP. As shown in (Figure 4A), the root system of each virus-inoculated plant was maintained in a single tube to allow clear observation of root changes upon TMV-GFP infection. On day 1 post-inoculation, no significant difference in root morphology were observed compared to healthy plants. By day 3, lateral root development at the root tips had stopped in infected plants relative to healthy controls. By day 5, overall lateral root growth was slower, and root system expansion had noticeably slowed. By day 7, the root systems of infected plants appeared thinner and weaker, with significantly less roots compared to healthy plants.

Changes in water level were monitored to assess the impact of viral infection on the transpiration rate of individual plants. On day 1 post-inoculation, the water level in tubes containing infected plants decreased by 1.23  cm more than in those with healthy plants. On 3, 5, and 7 d post inoculation, the decreases in water level were 0.31  cm, 0.12  cm, and 0.14  cm, respectively, relative to healthy plants (Figure 4B).

### Tracking the TMV-GFP infection in roots of *N. benthamiana*

**Figure 5.**
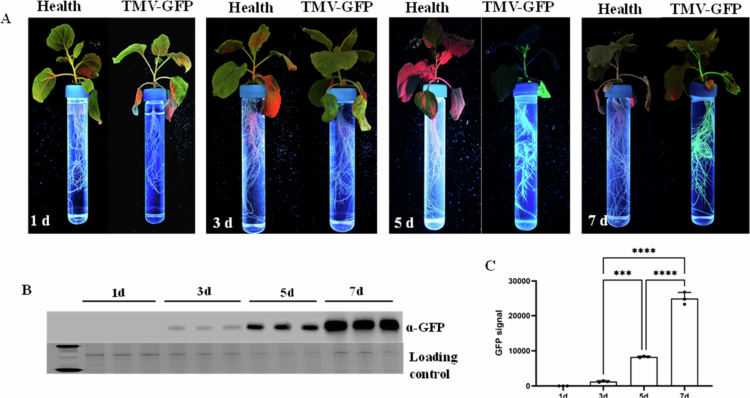
Distribution of TMV-GFP in the root system. (A) Dynamic infection of TMV-GFP in the root system at different days; (B) detection of TMV accumulation by Western blot; (C) quantitative analysis of GFP signals by Quantity One; *** *P *< 0.001, **** *P *< 0.0001.

Leaves of *N. benthamian* plants grown in hydroponical system were inoculated with TMV-GFP, and viral infection symptoms were observed at different days post inoculation. The results showed that GFP fluorescence in the roots of TMV-GFP-inoculated plants progressively increased over time (Figure 5A). Entire root systems of *N. benthamian*a were collected for quantitative analysis of viral accumulation with anti-GFP. The results indicated a statistically significant increase in viral accumulation in the root systems of TMV-GFP-inoculated plants over time (*P *< 0.001) (Figure 5B,C).

## Discussion

The root systems of plants provide crucial support for the plants by penetrating and anchoring themselves in the soil matrix. This enables the plants to withstand both biotic and abiotic stresses and maintain the spatial structure necessary for growth and development.^2^ Roots also act as absorption, transportation, and storing resources. Through special structures such as root hairs, they are able to effectively absorb water and mineral nutrients from the soil.^18^ These substances will then be transported through the vascular system to the above-ground organs. Moreover, the roots can also store the products of photosynthesis to regulate the overall resource allocation and energy metabolism of the plant. Furthermore, the roots can also store the products of photosynthesis, which helps regulate the overall resource allocation and energy metabolism process of the plant.^19^

Hydroponics, as a new type of plant cultivation method, has significant advantages over traditional soil cultivation systems in terms of root system research. First, roots are directly exposed to the nutrient solution, avoiding the need for destructive operations such as digging and washing, which can cause root damage. This enables nondestructive, real-time observation of roots, greatly improving the accuracy and reproducibility of experimental data.^20^ Second, the composition of the nutrient solution and environmental factors can be precisely controlled, avoiding interference from soil heterogeneity, thereby facilitating efficient analysis of the regulatory mechanisms underlying root morphological development and physiological metabolism.^21^ Particularly, hydroponics system offers some advantages for root research. It not only enables precise imaging of the three-dimensional movements of root tips,^22^ but also facilitates reproducible studies on suberization and water transport in poplar roots.^23^ Furthermore, it supports comparative analyzes of root phenotypes in hydroponically grown versus soil-grown peanuts, among other applications.^24^

Currently, research on the infection of plant viruses in roots remains limited. This study provides a novel experimental method for obtaining the complete root system of individual *N. benthamiana* plants. These results indicate that, compared with those under conventional cultivation in soil, seed germination in the hydroponic system showed cotyledon expansion within 5–7 d. whereas plants cultivated in soil showed cotyledon expansion concentrated within 4–5 d. Although the total number of seeds are different between hydroponic and soil conditions, the germination rates were calculated according to the following equation.

germinationrate(%)=(totalnumberofgerminatedseeds/totalnumberofsownseeds)×100. All experiments were set with three independent biological replicates to eliminate the influence of seed number differences. When the *N. benthamiana* seedlings reached the 4–5 leaf stage, individual plants were transferred to flat-bottomed test tubes, allowing each plant's root system to be maintained in a relatively controlled and independent environment. This setup not only facilitates the analysis of root exudates from individual plants but also enables the observation of plant responses under stress. After inoculating the *N. benthamiana* seedlings with TMV-GFP, overall lateral root development was slowed, root growth was significantly inhibited, and the number of roots was notably reduced. By continuously monitoring the height of the nutrient solution surface, the transpiration rate of virus-infected plants could be quantified based on the change in liquid level. The authors observed that the transpiration rate increased significantly immediately after virus inoculation but gradually decreased over time However, this is only an observational result, and its underlying regulatory mechanism requires further study.

The single-plant controlled hydroponic system established in this study provides efficient technical support for research on plant root‒virus interactions. In the future, this system can be extended to investigate the root infection mechanisms of various crop viruses. Moreover, the advantages of single-factor regulation and nondestructive observation can be harnessed to explore the regulatory effects of environmental factors on root‒virus interactions, which will lay a practical foundation for the green prevention and control of crop viral diseases and offer a methodological reference for related studies on plant‒pathogen interactions and stress physiology.
